# Membrane Interaction of Bound Ligands Contributes to the Negative Binding Cooperativity of the EGF Receptor

**DOI:** 10.1371/journal.pcbi.1003742

**Published:** 2014-07-24

**Authors:** Anton Arkhipov, Yibing Shan, Eric T. Kim, David E. Shaw

**Affiliations:** 1 D. E. Shaw Research, New York, New York, United States of America; 2 Department of Biochemistry and Molecular Biophysics, Columbia University, New York, New York, United States of America; Max Planck Institute for Biophysical Chemistry, Germany

## Abstract

The epidermal growth factor receptor (EGFR) plays a key role in regulating cell proliferation, migration, and differentiation, and aberrant EGFR signaling is implicated in a variety of cancers. EGFR signaling is triggered by extracellular ligand binding, which promotes EGFR dimerization and activation. Ligand-binding measurements are consistent with a negatively cooperative model in which the ligand-binding affinity at either binding site in an EGFR dimer is weaker when the other site is occupied by a ligand. This cooperativity is widely believed to be central to the effects of ligand concentration on EGFR-mediated intracellular signaling. Although the extracellular portion of the human EGFR dimer has been resolved crystallographically, the crystal structures do not reveal the structural origin of this negative cooperativity, which has remained unclear. Here we report the results of molecular dynamics simulations suggesting that asymmetrical interactions of the two binding sites with the membrane may be responsible (perhaps along with other factors) for this negative cooperativity. In particular, in our simulations the extracellular domains of an EGFR dimer spontaneously lay down on the membrane in an orientation in which favorable membrane contacts were made with one of the bound ligands, but could not be made with the other. Similar interactions were observed when EGFR was glycosylated, as it is in vivo.

## Author Summary

Epidermal growth factor receptor (EGFR) molecules are of central importance in cellular communication. Embedded in the cell membrane, these receptors bind epidermal growth factor (EGF) molecules outside the cell and translate this binding into specific biochemical signals inside the cell, which in turn trigger cell proliferation, migration, or differentiation. EGFR dysfunction has been implicated in a variety of cancers, and EGFR-targeting drugs are commonly used in cancer treatments. It has been widely assumed that the extracellular portion of an EGFR molecule protrudes perpendicularly from the cell membrane. In detailed, atomic-level computer simulations, however, we find that it lies down on the membrane, placing its EGF-binding site adjacent to the membrane surface. We further show that EGF may interact with EGFR in two distinct ways (with or without the involvement of the membrane). This may explain the experimental finding that an EGF molecule binds to EGFR more weakly at higher EGF concentration. This phenomenon, which is a manifestation of an underlying negative cooperativity, is an important but poorly understood characteristic of EGFR activity. In this study, we also model and analyze the glycan chains attached to EGFR, which are integral to its behavior in living cells.

## Introduction

The epidermal growth factor receptor (EGFR), a member of the Her (ErbB) family of cell-surface receptors, is critical to a variety of cellular processes and is implicated in the development of several forms of cancer and other diseases [1]–[3]. In normal cells, EGFR activation is initiated by the binding of extracellular ligands from the epidermal growth factor (EGF) family [4]–[7], giving rise to the formation of active EGFR dimers, which transmit intracellular signals. It was first shown 30 years ago [8]–[12] that the Scatchard plots of EGF binding to EGFR are nonlinear (concave up), which is indicative of heterogeneous binding affinity. It has been further suggested that the heterogeneity in EGFR ligand binding may play an important role in determining the signaling response to different ligand concentrations [12]–[14].

**Figure 1:**
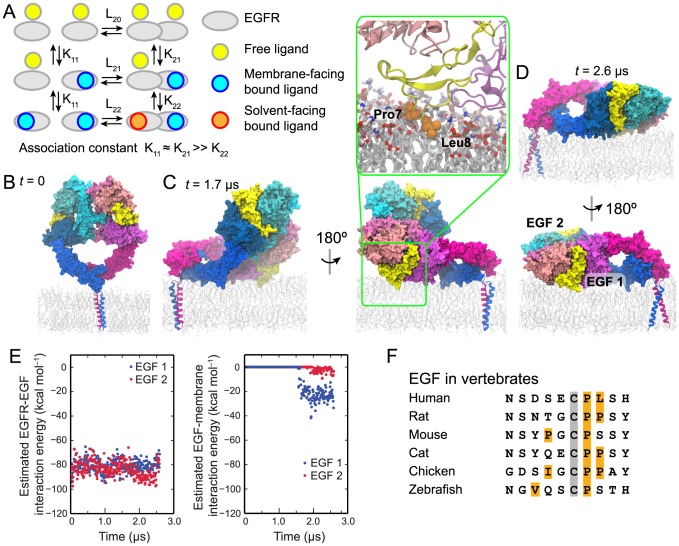
The ectodomain portion of the EGFR dimer lying on the membrane. (A) A schematic description of EGFR ligand binding. The basic scheme is taken from ref. [15], but we have color coded the ligands to distinguish the free, the membrane-facing bound, and the solvent-facing bound ligands according to the simulation findings. “L” and “K” are used to denote the association constants of EGFR dimerization and ligand binding, respectively. The negative cooperativity is reflected in K_21_≫K_22_. The ligands bound to monomers are assumed to face the membrane as found in previous simulations of EGFR monomers (ref. [26]; see the Discussion). (B–D) Simulation of an EGFR dimer construct consisting of the ectodomains and TM helices. One EGFR subunit is colored in shades of blue, the other in shades of red, and the two bound EGF ligands in yellow. In the initial state (B), the ectodomain dimer is standing upright, perpendicular to the membrane. In the course of the simulation, the ectodomain dimer approaches the membrane (C), permitting the formation of extensive interactions between one of the ligands and the membrane, resulting in the partial-resting orientation. The EGF side chains Pro7 and Leu8 (orange) penetrate deep into the membrane, reaching the lipid tails (inset). Later in the course of the simulation (D), the ectodomain dimer approaches closer to the membrane and lies flat on its surface, in the full-resting orientation. (E) The free energy of each ligand's interaction with its host receptor (EGF1, blue; EGF2, red) in a two-ligand EGFR dimer (left panel) and of its interaction with the membrane bilayer (right panel); calculations used the MM/GBVI method. (F) The N-terminal sequences of EGF in various vertebrate species and from a set of members of the human EGF family. The amino acids shown correspond to residues 1–10 of human EGF. The conserved Cys6 is marked in gray and hydrophobic residues in positions 1–8 are marked in orange. Hydrophobic and aromatic residues beyond position 8 are expected to be buried in the protein interior.

More recently, in a study conducted by Pike and colleagues [15], a characterization of EGFR ligand binding based on a simultaneous fitting of binding isotherms from cells with different levels of EGFR expression showed that EGFR ligand binding may be described by a simple model (shown in Fig. 1A). In this model, which is consistent with earlier results [16], negative cooperativity underlies the heterogeneity of EGFR ligand binding [15], [16]: The binding affinity of a ligand at one EGFR binding site is smaller when the other site is occupied).

The structural origin of this negative cooperativity has been unclear. The existence of two structurally distinct binding sites in the doubly liganded dimer of *Drosophila* EGFR (dEGFR) [17] is consistent with its binding cooperativity. In crystal structures of the doubly liganded human EGFR dimer, however, the two binding sites are structurally virtually identical [18], [19]. A recent investigation, based on structural and biochemical analyses [20], suggested that the ligand-binding cooperativity may be explained by a conformational change in the ectodomain dimer. Although it is very plausible that such a scenario explains some of the negative cooperativity in EGFR, it is not clear that it represents the only, or even the main, contribution.

Here we use molecular dynamics (MD) simulations to investigate the structural basis of the negative cooperativity in the ligand binding of human EGFR. We simulated the human EGFR ectodomain dimer anchored to a lipid membrane by EGFR transmembrane (TM) helices. In our simulations of both singly and doubly liganded ectodomains, the dimer began in an upright orientation, with the dimer's long axis perpendicular to the membrane, then spontaneously rotated and lay down on the membrane in such a way that one of the binding sites faced the membrane, while the other faced the bulk solvent. The ligand in the membrane-facing site developed extensive favorable interactions with the membrane, and our approximate free energy calculations suggest that these interactions contribute a significant fraction of the ligand binding free energy. These findings are consistent with Förster resonance energy transfer (FRET) experiments [21], [22], which showed that some EGFR-bound ligands are positioned within 40 Å of the membrane, while others are positioned beyond 70 Å. In further simulations of glycosylated EGFR ectodomains, we found that the ectodomain orientation and the membrane interaction of the bound ligand appear compatible with the in vivo glycosylation of EGFR.

Based on our simulation findings, we suggest that the negative cooperativity in human EGFR ligand binding may arise in part from broken symmetry between the two bound ligands in an orientation in which the ectodomain rests on the membrane. Specifically, the simulations showed that the membrane favorably interacts with ligands bound to EGFR ectodomains resting on the membrane, and the results suggest that the high-affinity binding to an unliganded EGFR dimer may be attributed to this previously largely overlooked ligand-membrane interaction (Fig. 1A). Such a structural explanation is supported by the experimental finding that when high-affinity ligand binding is abolished, the distance between bound ligands and the membrane increases [22]. The mechanism we propose here also offers a straightforward explanation of the observation that negative ligand-binding cooperativity in human EGFR is observed only when the receptor is embedded in the membrane.

## Results

### The two ligands bound to an EGFR dimer may interact with the membrane differently

We first simulated a dimer of the ectodomains (domains I, II, III, and IV) and the single-helix TM segment of EGFR. Based on available crystal structures [18], [19], [23], the ectodomains were prepared in the form of an EGF-bound, back-to-back symmetric dimer, and the TM helices were prepared in the form of a TM dimer, with the N-terminal GxxxG-like motifs (where G represents glycine, or another amino acid with a small side chain) as the dimer interface [24]–[26]. The ectodomains were initially positioned upright, approximately perpendicular to the membrane (Fig. 1B). In the simulation, the ectodomains lay down toward the membrane surface, and approximately 1.7 µs into the simulation, one of the bound ligands developed extensive interactions with the membrane (Fig. 1C) in a *partial-resting* orientation of the ectodomain dimer. Later in the course of the simulation, the ectodomains lay flat on the membrane, producing a *full-resting* orientation (Fig. 1D). The simulation observations are supported by FRET measurements [21], [22], which have indicated that the EGFR ectodomain dimer may rest on the membrane. The simulation indicates that such orientations of the ectodomains are made possible because the linker segments between the ectodomains and the TM helices are not fully rigid, as has been previously suggested [23]. We previously simulated EGFR ectodomain monomers tethered to membrane-embedded TM helices. Although the ectodomain is ligand-free in the simulations, it came to rest on the membrane from an upright orientation in such a way that, if it were ligand-bound, the ligand would be in contact with the membrane (Fig. 6A in ref. [26]).

Notably, the orientation of the ectodomain dimer with respect to the membrane broke the symmetry between its two bound ligands: One of the ligands (the *membrane-facing* ligand) but not the other (the *solvent-facing* ligand) was in contact with the membrane (Fig. 1C). Once the ectodomain dimer rested on the membrane, the membrane-facing ligand developed extensive favorable interactions with the lipids. In particular, the hydrophobic residues of the EGF ligand, such as Pro7 and Leu8, were found to enter the hydrophobic interior of the membrane's extracellular leaflet (Fig. 1C, inset). As discussed in detail in later sections, similar membrane interactions were also observed for one of the bound ligands in two other EGFR dimer simulations we performed.

Although it is challenging to accurately calculate biomolecular binding free energies in simulation, generalized Born models can often provide a rough estimate. We estimated the binding free energy of each bound ligand using the molecular mechanics/generalized Born volume integration (MM/GBVI) model (see Methods) [27]. We initially calculated what we refer to as the ligand-protein *interaction energy*, which is an estimate for the free energy if there is little change in the protein structure on binding. As shown in Fig. 1E, the simplest application of the MM/GBVI method yields an interaction energy of an EGF ligand with EGFR of ∼80 kcal mol^−1^. The ligand-membrane interaction energy contributes an additional ∼25 kcal mol^−1^ to the membrane-facing ligand but almost nothing to the solvent-facing one.

The EGF-EGFR interaction energy of over 80 kcal mol^−1^ is much higher than the experimental value of the EGF binding free energy (10–15 kcal mol^−1^ [15]). As noted above, however, the computational quantity does not include the conformational free energy cost incurred when EGFR adopts its ligand-bound conformation during EGF binding. Using the structures of the active [18], [19] and inactive [26] dimers, the MM/GBVI model estimates the cost of EGFR's transition to the ligand-bound conformation to be 89 kcal mol^−1^ per monomer in the EGFR dimer. This is qualitatively consistent with our previously reported simulations [26], in which the ligand-bound conformation was not stable without the EGF ligands, and it suggests that the favorable EGF-EGFR interaction is approximately canceled out by EGFR adopting the unfavorable ligand-bound conformation. Despite this apparently satisfactory cancellation, we suspect that the individual canceling terms may still be overestimates, and we regard the use of the generalized Born model in the present context as qualitative.

To assess whether interactions with the membrane could contribute significantly to the experimental ligand-binding free energy, we do not only look at the large (∼25 kcal mol^−1^) estimated binding energy, but we also look at how this energy compares to the estimated ligand-protein interaction energy. Since the protein-membrane energy is a substantial fraction even of the large ligand-protein interaction energy, the generalized Born model supports the conclusion that the membrane-facing ligand binds to the EGFR dimer with higher affinity than does the solvent-facing one.

Intriguingly, we found that the hydrophobic patch formed by Pro7 and Leu8 is largely conserved in vertebrate EGF molecules (Fig. 1F). Pro7 is especially well conserved in vertebrate EGF. Leu8 is less conserved, but this position is hydrophobic in the majority of vertebrate EGF members despite being solvent accessible.

### Interactions between the membrane and glycosylated EGFR

**Figure 2:**
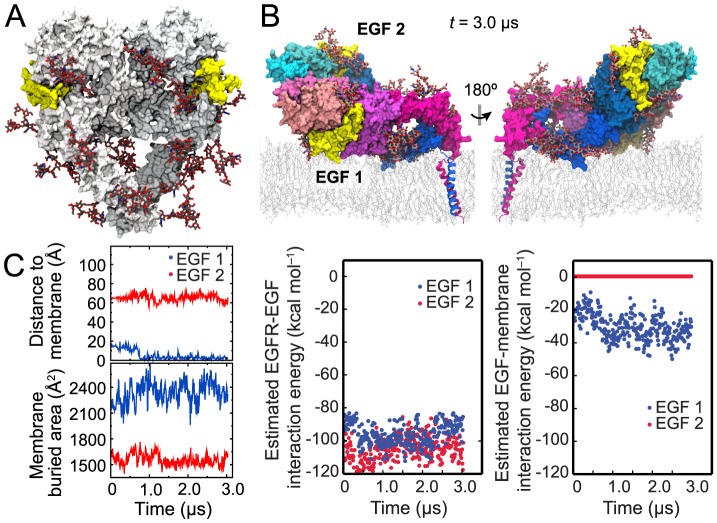
Simulation of fully glycosylated EGFR. (A) A fully glycosylated ectodomain dimer of EGFR. The BiS1F1, Man8, and Man6 glycans attached to EGFR are colored by atom type (gray for carbon, red for oxygen, and blue for nitrogen). (B) The conformation at the end of the simulation shown from two opposite directions. (C) Distance between the N terminus of each ligand (blue, membrane-facing ligand; red, solvent-facing ligand) and the membrane surface (see Methods) in the glycosylated-EGFR simulation, and total surface area of the ligand buried due to its interactions with the receptor and the membrane (left panels). Also shown are the results of the MM/GBVI calculations of the free energy of each ligand's interaction with its host receptor in the glycosylated two-ligand EGFR dimer (middle panel) and the results of similar calculations for each ligand's interaction with the membrane bilayer (the right panel). The membrane-facing ligand enjoys greater binding free energy, and thus higher binding affinity, than the solvent-facing one due to the additional energy conferred by the membrane interaction.

In vivo, human EGFR is glycosylated on the ectodomains [28], and 10 of the receptor's 12 potential glycosylation sites are found to be fully or partially occupied by a variety of large, branching glycans [29], [30]. It is conceivable that the relatively bulky glycans may preclude an EGFR ectodomain dimer from resting on the membrane. To test whether this is the case, we modeled and simulated an EGFR ectodomain–TM dimer system with full glycosylation (Fig. 2A).

We decorated the EGFR ectodomains at the 10 identified glycosylation sites [29], [30] with three types of glycans (BiS1F1, Man6, and Man8) that are common in EGFR glycosylation (see Methods, Fig. S1, and Table S1). The simulation shows that the glycosylation does not disrupt the ligand-membrane contacts (Fig. 2B) we observed in nonglycosylated EGFR. In the course of the simulation starting from the partial-resting orientation (Fig. 1C), the orientation of the ectodomains with respect to the membrane remained unchanged, with the membrane-facing ligand embedded in the membrane and the other ligand facing the solvent. The simulation showed that, in addition to interglycan interactions, the polar glycans interact extensively with the protein and the lipid head groups. The flexibility of glycans allowed the ectodomains to rest on the membrane. The glycans were found to be distributed adjacent to the protein surfaces, rather than protruding into the solvent (Fig. S2). The membrane-facing ligand of the glycosylated EGFR exhibited the same degree of membrane interactions in the simulation (Fig. 2C) as that of the nonglycosylated EGFR (as indicated by the values of *t* = 0 in Fig. 2C). In fact, the membrane embedding of the glycosylated-EGFR membrane-facing ligand was slightly deeper than that of the membrane-facing ligand in nonglycosylated EGFR.

We calculated that the membrane interaction contributes an additional estimated ∼30% to the membrane-facing ligand's MM/GBVI binding energy but virtually nothing to that of the solvent-facing ligand (Fig. 2C). We thus conclude that robust interaction between EGFR-bound ligands and the membrane, which may contribute to the heterogeneous ligand binding in an EGFR dimer, is accessible to glycosylated as well as nonglycosylated EGFR.

### The ligand in a one-ligand dimer interacts with the membrane

Having demonstrated that the ectodomains in a two-ligand EGFR dimer may rest on the membrane and that the two ligands may differ in their interactions with the membrane due to the ectodomain's orientation, we further simulated the one-ligand EGFR dimer. These simulations suggest that the ectodomains may rest on the membrane and that the ligand in a one-ligand EGFR dimer may also develop favorable interactions with the membrane, thus providing a structural model for high-affinity binding in the one-ligand EGFR dimer (Fig. 1A).

**Figure 3:**
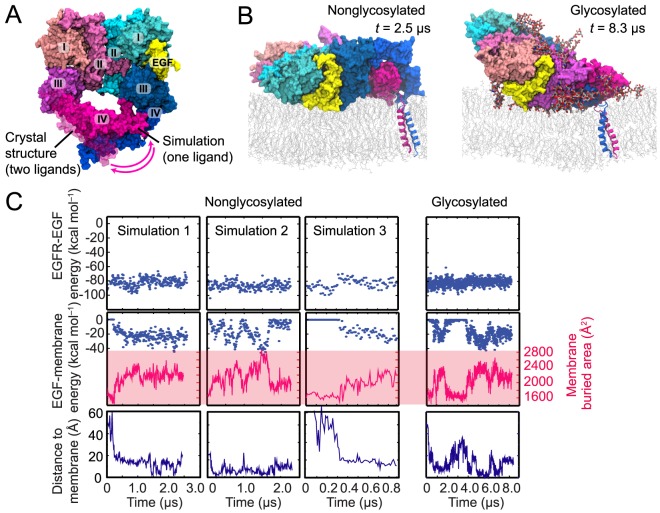
Simulations of the one-ligand dimer. (A) The conformation of the one-ligand ectodomain dimer obtained from a simulation employing the crystal structure of the two-ligand dimer (PDB entry 3NJP; ref. [23]), with the ligand removed from the red subunit, as a starting state. Domains I–IV and the EGF molecule are marked. The one-ligand dimer differs from the two-ligand dimer in the conformation of the domain IV of the red subunit. (B) The one-ligand dimer lying down on the membrane. The ligand bound to this dimer faces the membrane. Snapshots from simulations of the nonglycosylated and glycosylated dimers are shown. (C) The free energy of a ligand's interaction with its host receptor in a one-ligand EGFR dimer (upper panels) estimated using MM/GBVI, the strength of its interaction with the membrane bilayer (middle panels) estimated in the same way, and the distance between the ligand's N-terminus and the membrane (lower panels) in three independent simulations in which the receptors were not glycosylated and in one additional simulation in which they were. Also shown (middle panels) is the total surface area of the ligand buried due to its interactions with the receptor and the membrane. As indicated by these data, the additional free energy conferred by the ligand's membrane interaction is a significant fraction of its interaction energy with the receptor.

Because a crystal structure of a singly liganded ectodomain of an EGFR dimer is not yet available, we made a model based on the crystal structure of the two-ligand ectodomain dimer by removing one bound ligand from the crystal structure [26]. We here simulated the one-ligand ectodomain dimer in this conformation, connected with the TM segments, three times. In all three simulations, the ectodomain dimer, which was initially in an upright orientation, spontaneously lay down on the membrane (Fig. 3B), allowing the ligand to come into contact and develop extensive interactions with the membrane. We again calculated the MM/GBVI energy of the ligand's interaction with the membrane (Fig. 3C). The results suggest that the membrane interaction is energetically favorable, and that the free energy increase associated with the ligand's membrane interaction is a significant fraction of the free energy arising from its interaction with EGFR.

The favorable nature of the ligand-membrane interaction strongly suggests that the membrane-facing binding sites are associated with high-affinity binding. This notion is notably supported by the observation from FRET experiments that abolishing the high-affinity binding leads to a significant increase in the average distance between the ligands and the membrane [22]. Assuming a thermodynamically equilibrated system, in one-ligand EGFR dimers the ligands predominantly occupy the high-affinity sites facing the membrane (Fig. 1A).

We also performed a similar simulation of a fully glycosylated, one-ligand ectodomain dimer attached to the TM segments. Starting form an upright conformation, the ectodomain dimer again spontaneously lay down, with its bound ligand coming into contact with the membrane (Fig. 3B and 3C). This simulation suggests that a glycosylated one-ligand dimer may also prefer to rest on the membrane in such a way that its bound ligand faces the membrane, and that the ligand-membrane interactions are energetically favorable

### The two-ligand ectodomain dimer remains symmetric in the simulations

**Figure 4:**
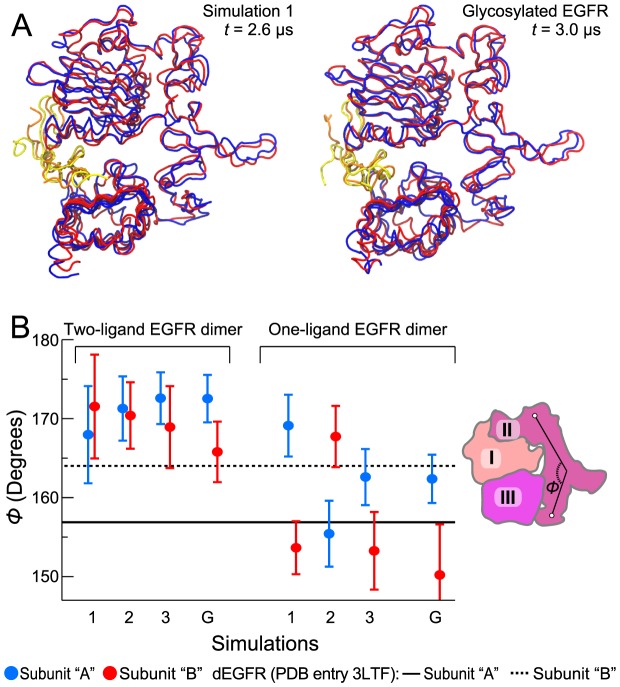
The two-ligand ectodomain dimer remaining symmetric in the simulations. (A) The two subunits of the ectodomain dimer, as observed at the end of one of the simulations without glycosylation (left) and at the end of the simulation with full glycosylation (right), superimposed using the Cα atoms of domains I–III for reference. The EGFR ectodomains I–III are shown in blue and red, and the EGF molecules bound to them are shown in yellow and orange, respectively. (B) Domain II maintaining the same conformation in both subunits of the two-ligand EGFR dimer. Angle *Φ* (the angle formed by Cα atoms of residues 194, 239, and 296 in EGFR, or 189, 235, and 289 in dEGFR) characterizes the bending of domain II. This angle is different in each of the two subunits of the asymmetric two-ligand dEGFR dimer (the solid and dashed black lines; PDB entry 3LTF). The average angles in the simulations of two- and one-ligand EGFR dimers (Figs. 2, 3, and 5; labels refer to nonglycosylated EGFR simulations 1, 2, and 3, and glycosylated EGFR simulation G) are shown for each of the two subunits in blue and red (error bars correspond to the standard deviation). The angle *Φ* is illustrated in the schematic of domains I, II, and III of EGFR on the right.

**Figure 5:**
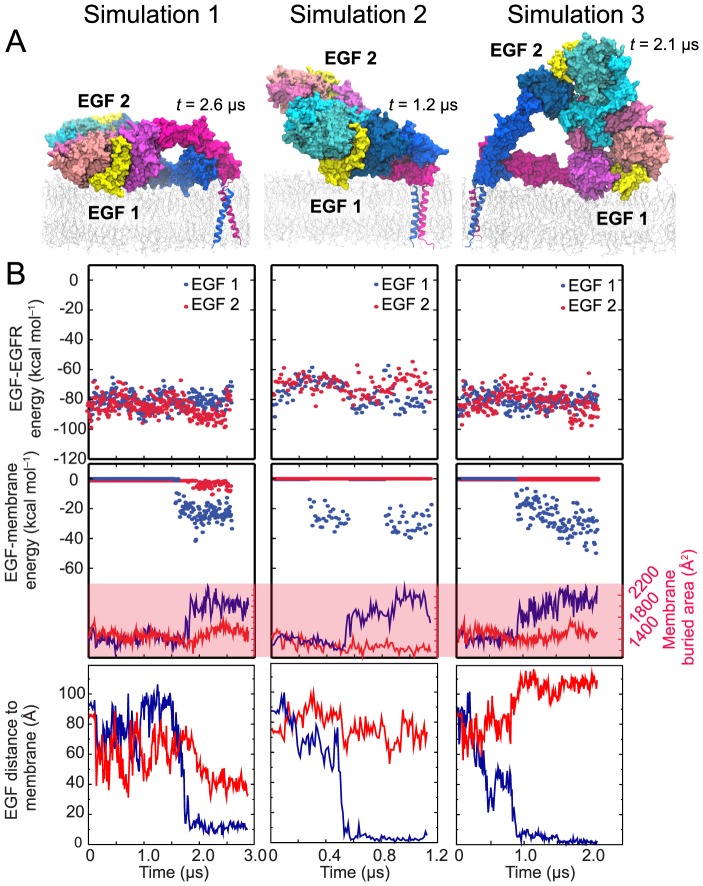
Ligand-membrane interaction in simulations of the two-ligand EGFR dimer. (A) Snapshots from the endpoints of the simulations. The ectodomain dimers lie down on the membrane surface in a variety of ways; in each case, however, only one of the two ligands establishes strong interactions with the membrane. (B) The free energy of each ligand's interaction with its host receptor in a two-ligand EGFR dimer (upper panels) estimated using MM/GBVI, the strength of its interaction with the membrane bilayer (middle panels) estimated in the same way, and the distance between its N-terminus and the membrane (lower panels) in three independent simulations. In the middle panels, the surface area of each ligand buried by the membrane is plotted. As shown, the membrane-facing ligand (blue) enjoys greater binding free energy, and thus higher binding affinity, than the solvent-facing one (red) due to the additional energy conferred by the membrane interaction.

A simulation study of EGFR [22] has previously suggested that ectodomain interactions with the membrane may be at the root of the observed negative cooperativity of ligand binding. It was further suggested that the negative cooperativity may arise from the ectodomain's transition to a dEGFR-like asymmetric conformation, induced by interactions with the membrane. Although our simulations also suggest the important role of EGFR ectodomain interactions with the membrane, our simulations did not show a robust transition from a symmetric to an asymmetric conformation in the ectodomain dimer. Fig. 4A shows that, other than minor deviations due to the inherent flexibility of the loop regions, the dimer's two ectodomain subunits were nearly conformationally identical in our simulations. In particular, the conformations of domain II in the two subunits are highly similar, whereas in the dEGFR dimer the domain II is straight in one subunit and bent in the other, which ultimately leads to the different conformations of the two binding sites. This is illustrated in Fig. 4B, where the angle characterizing the bending of domain II is plotted. The angles of the two EGFR subunits were approximately the same in our simulations of the two-ligand dimer, much as they are in crystal structures [18], [19]. Our MM/GBVI calculation supports the notion that the two receptors of the two-ligand EGFR dimer maintain similar binding-site conformations while resting on the membrane: The two ligands have comparable MM/GBVI interaction energies with the receptors (Fig. 5B), including cases in which the receptors are glycosylated (Fig. 2C).

**Figure 6:**
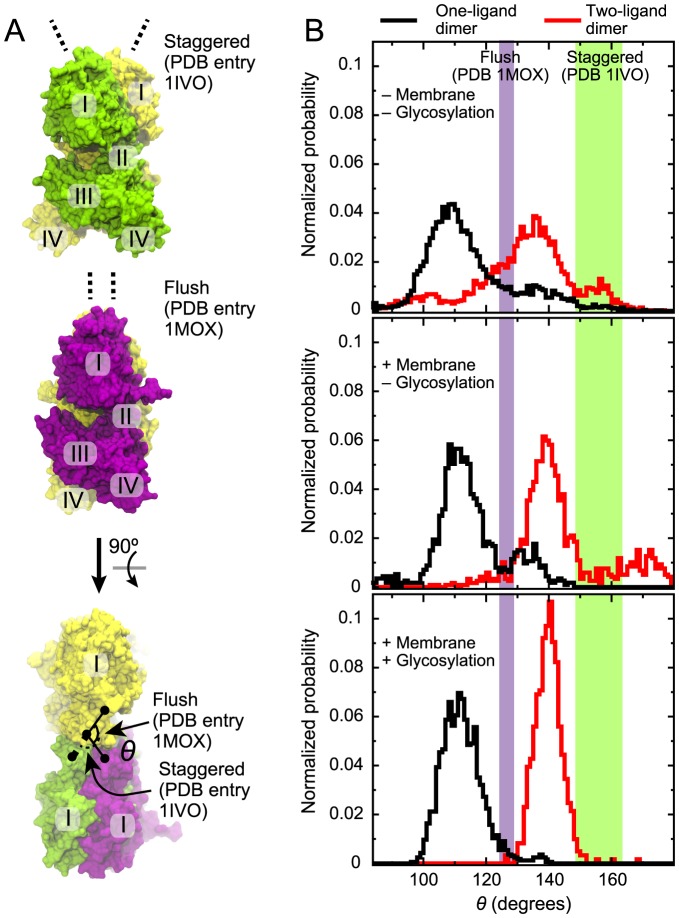
“Staggered” and “flush” conformations of the extracellular dimers. (A) The staggered and flush conformations [20] are observed in PDB entries 1IVO and 1MOX, respectively. These two conformations are shown at the top and in the middle. At the bottom, the yellow subunits of both crystal structures are superposed and the view is from above (relative to the other two images). The conformations can be distinguished by the angle *θ* formed by the Cα atoms of Ile190 and Pro204 of one subunit and Pro204 of the other. (B) Distributions of *θ* observed in simulations of the one- (black) and two-ligand (red) EGFR dimers. Data from the simulations of ectodomains in solution, reported in ref. [26], and data from simulations of nonglycosylated and glycosylated EGFR constructs with the membrane, which are reported in the present study, are shown from top to bottom, respectively. Values of *θ* from the crystal structures are indicated. Two slightly different *θ* values are obtained for each crystal structure, because the structures are not exactly symmetric; the spaces between these values are shown as colored bands.

On the other hand, for the one-ligand dimer, which assumes asymmetric conformations, the angles differ significantly between the two subunits. Similarly, in our simulations the average root-mean-square deviation (RMSD) of the Cα atoms of domains I, II, and III between the two subunits was significantly lower for the two-ligand ectodomain dimer (2.7±0.3 Å when glycosylated and 2.6±0.5 Å when nonglycosylated) than for the one-ligand dimer (4.4±0.2 Å when glycosylated and 4.7±0.3 Å when nonglycosylated).

### Variation in the orientations of the ectodomain dimer

We performed three independent simulations of the two-ligand, nonglycosylated EGFR dimer. As discussed above, in one of these simulations, the ectodomain dimer first assumed a partial-resting orientation and eventually lay flat on the membrane (Fig. 1; also shown as Simulation 1 in Fig. 5). In another simulation (Fig. 5A, Simulation 2), the system arrived at a similar partial-resting orientation and remained there to the end of the simulation. In the third simulation (Fig. 5A, Simulation 3), the ectodomain dimer was found to rest sideways on the membrane surface. What is common to all three simulations, however, is that only one bound ligand made extensive contact with the membrane (Fig. 5B, upper panels) despite the variation in the orientation of the ectodomain dimer. The buried surface area of the membrane-facing ligand was consistently greater than that of the solvent-facing ligand (Fig. 5B): 2,300±100 Å^2^ versus 1,600±100 Å^2^. A substantial portion (up to 200 Å^2^) of the approximately 700-Å^2^ difference is due to the embedding of Pro7 and Leu8 in the membrane. Our MM/GBVI calculations also consistently suggest that ligand-membrane interactions contribute a significant fraction to the free energy of ligand-receptor binding (Fig. 5B).

Earlier FRET measurements indicated that EGF bound to EGFR dimers falls into two groups: one in which the bound ligand is close to the membrane, and another in which it is farther away. Specifically, the FRET results showed that the N termini of the EGF molecules in the “close” group are no more than 35–40 Å from the membrane, and the N termini of the EGF molecules in the “far” group are no closer than 69–71 Å from the membrane [21], [22]. Our simulation results (Figs. 2C and 5B) agree with these data (see the description of the distance measurements in the Methods section) and the simulation conformations are similar to those in the structural model proposed by Kästner et al. based on their FRET results [31]. In all of our simulations, the membrane-facing ligands were close to the membrane surface (∼10 Å), and thus belong to the former population. This population may also include the solvent-facing ligands in cases in which the ectodomain dimer rests flat on the membrane, such as at the end of Simulation 1, where the membrane distance is ∼40 Å for the solvent-facing ligand. The latter population, on the other hand, may consist of the solvent-facing ligands in dimers such as those observed in Simulations 2 and 3, as well as those in the upright dimers (with distances of ∼80–120 Å).

Combining the observations from the simulations with those from the FRET measurements, we suggest that it is unlikely that the negative cooperativity of ligand binding can be attributed to a single specific orientation of the ectodomain dimer. We instead suggest that the cooperativity is associated with an ensemble of ectodomain-dimer orientations, with the shared feature that the high-affinity ligand binding occurs at the membrane-facing binding site. This provides a straightforward explanation for the experimental observation that abolishing high-affinity ligand binding increases the average ligand-membrane distance [22]. Additionally, our simulations showed that free EGF molecules may interact favorably with and be attached to the membrane (Fig. S3). This simulation finding, combined with the observation that Spitz ligands (which bind to dEGFR) need to be palmitoylated (and thus attached to membrane) to activate dEGFR in vivo [32], raises the possibility that the ligand-binding process of EGFR may occur at the membrane surface.

## Discussion

Our simulations suggest that an EGFR ectodomain dimer may rest on the membrane, and that the interaction between a bound ligand and the membrane may lead to a breaking of the symmetry between the two ligands, thus contributing to the negative cooperativity of EGFR ligand binding (Fig. 1A). Our investigation is in part inspired by the FRET measurements of ligand distance from the membrane; based on these results, the orientation of EGFR ectodomains relative to the membrane was suggested to affect ectodomain conformations and give rise to the negative cooperativity [21], [22], [31]. The mechanism we propose here is particularly supported by the FRET finding that abolishing high-affinity ligand binding leads to a significant increase in the average distance between EGFR-bound ligands and the membrane [22]. Our simulations of glycosylated EGFR (to our knowledge the first simulations of a fully glycosylated receptor) showed that the mechanism we propose is compatible with EGFR glycosylation: A glycosylated ectodomain dimer may also rest on the membrane, and the attached glycans do not preclude interactions between the EGFR-bound ligand and the membrane.

In this investigation, we have largely focused on EGFR dimers because they are central to the negative cooperativity of EGFR ligand binding [14]. EGFR monomers may also bind ligands at a high affinity comparable to that of EGFR dimers [15], but the ectodomain structure of the ligand-bound EGFR monomer has not yet been resolved. In previous MD simulations, we showed that an EGFR monomer is similar to an EGFR dimer in that its ectodomains also rest on the membrane in a way that would allow membrane contact with the bound ligand [26]. From this observation, which is independent of any specific conformation of the ectodomains, it may be inferred that the high affinity of ligand binding in EGFR monomers could also be explained by favorable interactions between the membrane and the bound ligands.

Our simulations suggest that the ectodomains of an EGFR dimer may rest on the membrane and that a bound EGF ligand may be in direct and energetically favorable contact with the membrane. Our earlier simulations also suggest that EGFR monomer ectodomains may also rest on the membrane [26]. This does not imply, however, that the ectodomains are fixed on the membrane in well-defined orientations. It is likely that, on a timescale much longer than our simulations, the ectodomains convert from one orientation to another in a dynamic equilibrium. While the orientations in which the ectodomains rest on the membrane may predominate, the ectodomains likely access the other orientations that could be crucial to the process of ligand binding or EGFR dimerization.

A recent study [20] proposed that a conformational change from the so-called “flush” to the “staggered” arrangement between the two extracellular subunits in an EGFR dimer (Fig. 6A) may be at the root of the binding cooperativity of EGFR. While such a binding-cooperativity mechanism differs from the mechanism we propose here, these two mechanisms are not mutually exclusive. In agreement with the finding of Liu et al. [20] based on crystal structures, our simulations show that the two-ligand EGFR dimer prefers the staggered conformation and that the one-ligand and ligand-free EGFR dimers prefer the flush conformation [26]. Intriguingly, the ectodomain interaction with the membrane and the glycosylation of EGFR appear to strengthen this trend (Fig. 6B). From this observation, we suggest that the membrane may be of critical importance to the negative cooperativity of EGFR ligand binding, not only for its asymmetric interactions with the bound ligands, but also for its effect on the accessible conformational space of the ectodomain dimers. Further investigation is certainly needed to quantify the contribution of the conformational dynamics of the ectodomains and the contributions of ligand-membrane interactions to the ligand-binding cooperativity of EGFR. Further investigation would also be needed to clarify whether the membrane interactions of the ectodomains have any role in autoinhibition. We have not addressed this question, but we have previously shown that the membrane interactions of the EGFR kinase domain do play an autoinhibitory role [25], [26].

Experiments have shown that the ligand-binding cooperativity of EGFR is apparently missing for isolated EGFR dimer ectodomains in solution [12]. It was shown that the negative cooperativity may be partially recovered when the membrane is included in experiments of EGFR ectodomains attached to the TM helices [33]. Our suggested mechanism for the negative binding cooperativity, in which the membrane plays a central role, offers a straightforward explanation for these findings. If the asymmetry between the bound ligands in an EGFR dimer, and thus the binding cooperativity, is indeed associated with the difference in the interactions of bound ligands with the cell membrane, the absence of the membrane would naturally eliminate the binding cooperativity. Likewise, the lack of cooperativity for detergent-solubilized EGFR [34] may be explained by the absence of an extended membrane capable of interacting with EGFR-bound ligands. It has been shown that mutations at the intracellular domains of EGFR yield nearly linear Scatchard plots [33]. Although these Scatchard plots could reflect a weakened negative cooperativity due to these mutations, and thus suggest that the root of the negative cooperativity may lie beyond the ectodomains and the membrane, there is an alternative explanation: that the dimerization prior to ligand binding, which is a prerequisite of the binding cooperativity [15], was weakened, leading to both a near-linear Scatchard plot and a difficulty in using the plot to reliably quantify binding cooperativity [20].

Our investigation of the relationship between the EGFR ectodomains and the cell membrane using atomistic, long-timescale MD simulations suggests a structural mechanism for the negative cooperativity of ligand binding of EGFR dimers; in this mechanism, the ectodomains may rest on the membrane, and the presence of the membrane may break the symmetry between the two binding sites. These results add further support to the emerging view that interactions between EGFR and the membrane play a central role in many aspects of the regulation of EGFR signaling [25], [26], [34]–[36].

## Methods

The simulations were performed on a special-purpose supercomputer, Anton [37], using the Amber ff99SB-ILDN [38]–[40] force field, combined with the ff99SB* backbone correction [41] for proteins, the CHARMM C36 force field [42] for lipids, and TIP3P [43] as the water model. The simulated systems were solvated in water with 0.15 M NaCl, with residue protonation states corresponding to pH 7. Additional Na^+^ ions were included to neutralize the net charges of the proteins (−3 for the extracellular domains of each EGFR, −4 for each EGF ligand) and the POPS lipids. As an equilibration stage, the protein backbone atoms were first restrained to their initial positions using a harmonic potential with a force constant of 1 kcal mol^−1^ Å^−2^. The force constant was linearly scaled down to zero over at least 50 ns. Simulations were performed in the NPT ensemble with T = 310 K and P = 1 bar using the MTK algorithm [44] with 20-ps relaxation time. Water molecules and all bond lengths to hydrogen atoms were constrained using M-SHAKE [45]. The simulation time step was 1 fs for the equilibration stage and 2 fs for production simulations; the r-RESPA integration method was used, with long-range electrostatics evaluated every 6 fs [46].

The glycosylation of EGFR was modeled based on the mass-spectrometry analysis of the CL1-0 cell line [30], which is broadly consistent with similar analysis on CL1-5 and A431 cell lines [29], [30]. Since EGFR glycan attachments in the cell are very diverse—for every glycosylation site there is a large number of different glycan types that can be attached to it—we chose glycans among the most commonly found at the specific sites. These three common types are BiS1F1, Man6, and Man8 (Fig. S1 and Table S1). The glycan structures for the initial models were obtained using the Glycam web service [47] and then adjusted in VMD [48] to avoid clashes with protein and membrane. The simulations were performed with the GLYCAM06 force field [49] applied to the glycans.

The simulated systems included the ectodomain–TM dimers with two EGF molecules bound (three simulations of 2.6, 1.2, and 2.1 µs; ∼315,000 atoms) and with one EGF molecule bound (three simulations of 2.5, 2.3, and 0.9 µs; ∼300,000 atoms), a two-ligand glycosylated ectodomain–TM dimer (3.0 µs; ∼310,000 atoms), a one-ligand glycosylated ectodomain–TM dimer (8.3 µs; ∼300,000 atoms), and a single EGF molecule (see SI; two simulations of 8.9 and 8.3 µs; ∼62,000 atoms); a membrane was included in every case. Each system is set up such that each dimer is at least 25 Å from its periodic image.

The model membrane consisted of POPC lipids, with 30% (molar) POPC randomly replaced by POPS in the intracellular leaflet of the bilayer (only for the ectodomain–TM simulations) to approximately mimic the charge distribution in the cellular membrane [26], [50]. Modeling, analysis, and visualization were performed using VMD [48].

The distance between the EGF N terminus and the membrane, namely the distance from the N terminus to the plane through the phosphates of the extracellular lipid layer, was computed in a manner consistent with the FRET measurements [22].

The EGF-EGFR interaction energy estimation was based on the molecular mechanics/generalized Born volume integration (MM/GBVI) model [27] and performed using MOE software (Chemical Computing Group) [51]. The EGF-receptor binding energy was calculated for each snapshot from the difference of the energy of the EGF-receptor complex and the sum of isolated EGF and receptor energies. The EGF-membrane energy was calculated analogously. The conformational free energy of EGFR extracellular dimers was estimated based on the published coordinates of the full-length ligand-bound and ligand-free EGFR dimers [26] after energy minimization. Our calculations included domains I, II, III, and IV. The MM/GBVI energy is −34287.4 kcal mol^−1^ for the ligand-free dimer and −34110.2 kcal mol^−1^ for the ligand-bound dimer (the EGF ligands were not included in the calculation), and thus the conformational free energy cost for each monomer is 88.6 kcal mol^−1^.

## Supporting Information

Figure S1 **Schematic representation of the three types of glycans used in simulation.** The glycans BiS1F1, Man8, and Man6 [30] were attached to EGFR glycosylation sites as described in Table S1. The schematics shown represent the specific choices made for simulation; experimentally, there is ambiguity in terms of which branch some sugar rings belong to (e.g., NeuAc in BiS1F1). The attachment to the protein is on the right.
(EPS)

Figure S2 **Radius of gyration of all the glycan attachments and of the EGFR EC domains in simulations of glycosylated EGFR.** The plots on the top show radius of gyration computed for all 20 glycan attachments (excluding the EGFR and EGF proteins), and those on the bottom show radius of gyration for only the protein component of the EGFR ectodomains. In our initial models the glycan attachments protrude from the protein into the solvent—hence the relatively large initial radius of gyration. In simulation, however, the glycans engage in extensive interactions with the protein surface, resulting in a more compact overall arrangement. This is evidenced by a significant drop in the radius of gyration. The plots on the bottom show that this drop is not due to conformational changes in the protein, since the protein radius of gyration does not change significantly.
(EPS)

Figure S3 **Interactions between free EGF and the membrane.** (A) Distribution of the position of the EGF's center along the membrane normal (“*z*-axis”) relative to the center of the membrane is shown here. The panel includes data from two independent simulations (black and red, each over 8-µs long). Because the simulated systems are periodic, the EGF can switch from one side of the membrane to the other by crossing the periodic boundary. To account for this, the distributions over the “+*z*” and “−*z*” directions are combined into one distribution in the positive direction. (B) The simulation setup is shown here. EGF is initially placed away from the membrane, but it quickly attaches to the membrane and occasionally inserts the hydrophobic residues Pro7 and Leu8 into the membrane interior, as illustrated. Positions of the EGF center for the snapshots shown are projected on the plot. The shift between the peaks of the red and black distributions in (A) are due to the differences in the most frequently observed orientation of EGF with respect to the membrane in the two independent simulations. The upper peak corresponds to the orientation in which the long axis of EGF is perpendicular to the membrane, as illustrated in (B), and the lower peak corresponds to a different orientation, in which the long axis of EGF is parallel to the membrane.
(EPS)

Table S1 **Glycosylation of EGFR in simulations.** Among the 12 potential N-glycosylation sites of EGFR, two are not glycosylated (N104 and N172). In our simulation study, we attached one of three types of glycans—BiS1F1, Man6, or Man8—to each asparagine side chain of the 10 remaining sites.
(DOCX)

Text S1 **In the supporting information text and figures, simulations of EGF molecules interacting with the extracellular membrane are discussed.** Also discussed, in the SI Figures, are chemical and conformational details of the glycans attached to EGFR in our simulations.
(DOCX)
