# Supplementary material for: Membrane Interaction of Bound Ligands Contributes to the Negative Binding Cooperativity of the EGF Receptor
Source: PLoS Comput Biol. 2014 Jul 24;10(7):e1003742. doi: 10.1371/journal.pcbi.1003742 (PMC4109842; doi:10.1371/journal.pcbi.1003742)
Supplement: Table S1 — Glycosylation of EGFR in simulations. Among the 12 potential N-glycosylation sites of EGFR, two are not glycosylated (N104 and N172). In our simulation study, we attached one of three types of glycans—BiS1F1, Man6, or Man8—to each asparagine side chain of the 10 remaining sites. (DOCX) [file pcbi.1003742.s004.docx]

| Glycosylation site | Glycan attachment |
| --- | --- |
| N32 | BiS1F1 |
| N104 | None |
| N151 | BiS1F1 |
| N172 | None |
| N328 | Man8 |
| N337 | Man8 |
| Glycosylation site | Glycan attachment |
| N389 | BiS1F1 |
| N420 | Man6 |
| N504 | BiS1F1 |
| N544 | Man8 |
| N579 | BiS1F1 |
| N599 | Man8 |
