# Supplementary material for: Membrane Interaction of Bound Ligands Contributes to the Negative Binding Cooperativity of the EGF Receptor
Source: PLoS Comput Biol. 2014 Jul 24;10(7):e1003742. doi: 10.1371/journal.pcbi.1003742 (PMC4109842; doi:10.1371/journal.pcbi.1003742)
Supplement: Text S1 — In the supporting information text and figures, simulations of EGF molecules interacting with the extracellular membrane are discussed. Also discussed, in the SI Figures, are chemical and conformational details of the glycans attached to EGFR in our simulations. (DOCX) [file pcbi.1003742.s005.docx]

Supporting Information

Membrane Interaction of Bound Ligands Contributes
to the Negative Binding Cooperativity
of the EGF Receptor

# SI Results

## Free EGF may be preferentially found on the membrane surface

To further investigate the role of the membrane in EGFR ligand binding, we performed additional simulations of free EGF in the presence of the membrane. These simulations show that EGF molecules preferentially attach to the membrane surface. Initially placed in the bulk solvent, EGF tended to stay attached to the membrane after encountering it through diffusion. Only occasionally did the EGF detach from the membrane and return to the bulk solvent (Fig. S3A). In a substantial portion (30–50%) of the simulation time, an EGF molecule was anchored to membrane by the hydrophobic residues Pro7 and Leu8, which were buried in the interior of the membrane; this is reminiscent of the way an EGFR-bound, membrane-facing ligand interacts with the membrane (Fig. 1). Moreover, when the Pro7 and the Leu8 were buried in the membrane, the EGF molecule was oriented with respect to the membrane in a way similar to that of an EGFR-bound, membrane-facing ligand, so that the long axis of EGF was approximately perpendicular to the membrane plane (Fig. S3B).

These observations suggest that, although EGF molecules are known to be soluble [1], they may interact favorably with the membrane, and that the residues Pro7 and Leu8 may play an important role in mediating such interactions.

# SI References

1. Harris RC, Chung E, Coffey RJ (2003) EGF receptor ligands. *Exp Cell Res* 284(1): 2–13.
